# Hemoglobin Subunit Theta 1 Promotes Proliferation by Reducing Reactive Oxygen Species in Lung Adenocarcinoma

**DOI:** 10.3390/cancers15235504

**Published:** 2023-11-21

**Authors:** Kyungho Kim, Eun-Young Choi, Hye-Mi Ahn, Dong-Gun Kim, Youn-Jae Kim

**Affiliations:** Targeted Therapy Branch, Division of Rare and Refractory Cancer, Research Institute, National Cancer Center, Goyang 10408, Republic of Korea

**Keywords:** lung adenocarcinoma, HBQ1, reactive oxygen species

## Abstract

**Simple Summary:**

Lung adenocarcinoma, a leading cause of cancer-related deaths, poses treatment challenges. This study explores the role of HBQ1 in the disease. Elevated HBQ1 expression in adenocarcinoma tissues correlates with poor survival. Functional experiments reveal HBQ1 as an oncogene, promoting cell proliferation, and as an antioxidant, reducing reactive oxygen species levels. In vivo studies support HBQ1’s role in tumor growth, suggesting it as a potential therapeutic target. These findings highlight HBQ1’s crucial involvement in lung adenocarcinoma progression, proposing its use as a diagnostic marker and therapeutic target.

**Abstract:**

Lung adenocarcinoma is a crucial contributor to cancer-related mortality; however, effective treatments remain challenging. The present study aimed to investigate the role of hemoglobin subunit theta 1 (HBQ1), an α subunit of hemoglobin whose expression has recently been reported in non-erythroid cells, in lung adenocarcinoma. Comparative analysis showed that HBQ1 expression was significantly higher in lung adenocarcinoma tissues compared to normal lung tissues. Moreover, high HBQ1 expression was correlated with unfavorable overall survival and progression-free survival in patients, highlighting its potential as a prognostic marker. Our functional experiments revealed that when overexpressed, HBQ1 acts as an oncogene, enhancing cell proliferation, whereas HBQ1 knockdown inhibits it. Additionally, HBQ1 exhibited antioxidant properties by reducing basal reactive oxygen species levels, playing a crucial role in lung adenocarcinoma progression. These findings emphasize the critical role of HBQ1 in driving tumor growth and progression in lung adenocarcinoma. Our in vivo studies further supported the role of HBQ1 in lung adenocarcinoma. HBQ1 knockdown resulted in the inhibition of lung adenocarcinoma growth, demonstrating the potential of HBQ1 as a therapeutic target. Our findings highlight the importance of HBQ1 in lung adenocarcinoma and suggest its potential as both a diagnostic marker and a molecular target for therapeutic interventions.

## 1. Introduction

Lung cancer is one of the leading causes of cancer-related deaths worldwide, with non-small cell lung cancer (NSCLC) accounting for approximately 83% of all lung cancer cases [1,2,3]. Among the NSCLC subtypes, lung adenocarcinoma is one of the most commonly diagnosed, representing approximately 50% of NSCLC cases [4,5]. Despite advances in diagnosis and treatment, the prognosis of lung cancer remains poor; thus, further research is necessary in order to improve patient outcomes, particularly in those with lung adenocarcinoma cases. A comprehensive understanding of the molecular mechanisms underlying lung adenocarcinoma development and progression is crucial for the identification of novel therapeutic targets and the development of effective treatment strategies.

Hemoglobin is exclusively expressed in erythrocytes [6,7]. In humans, hemoglobin consists of two α-globin subunits and two β-globin subunits, forming a tetrameric structure. Its primary function is to transport oxygen from the lungs to various tissues in the body [8,9]. However, recent studies have revealed that hemoglobin is also expressed in non-erythroid cells, such as neurons [10,11,12], retinal cells [13,14,15], alveolar epithelial cells [16,17,18], endometrium [19,20], kidney mesangial cells [21,22], hepatocytes [23,24], and macrophages [25,26]. Furthermore, hemoglobin is differentially expressed in solid tumors, including breast, ovarian, and colorectal cancer [27,28,29]. A recent study has shown that hemoglobin subunit alpha 1 (HBA1) and hemoglobin subunit beta (HBB) levels were significantly elevated in patients with ovarian cancer compared to healthy patients [30]. These findings indicate that while hemoglobin primarily functions as an oxygen carrier in erythrocytes, emerging evidence suggests that it may also play diverse roles in cancer development and progression. Hemoglobin subunit theta 1 (HBQ1), an α-globin gene, is expressed exclusively in human fetal erythroid tissues and is, therefore, not expressed in adult erythroid tissues [7,31,32]. Recent studies have detected HBQ1 expression in non-erythroid cells, such as alveolar epithelial cells [18]. However, the functional role of HBQ1 expression in cancer biology, particularly in the development and progression of lung adenocarcinoma, remains to be elucidated.

Reactive oxygen species (ROS) are chemically reactive oxygen-containing molecules that are naturally generated as byproducts of cellular metabolism [33,34]. ROS arise during essential cellular processes such as mitochondrial respiration, inflammation, and immune responses [35,36]. They serve as crucial signaling molecules, contributing to processes such as cell apoptosis and differentiation [34,37,38,39]. However, excessive ROS accumulation induces oxidative stress, resulting in damage to DNA, proteins, and lipids [37,40,41]. ROS accumulation is closely associated with a range of diseases, including cancer, neurodegenerative disorders, cardiovascular diseases, and aging [42,43,44].

The present study aimed to investigate the role of HBQ1 in lung adenocarcinoma to expand the treatment strategies of the disease using both in vitro and in vivo experiments.

## 2. Materials and Methods

### 2.1. Antibodies and Reagents

The following primary antibodies were used: anti-HA (#3724; Cell Signaling Technology, Danvers, MA, USA), anti-HBQ1 (1:1000; 19997-1-AP; Proteintech, Chicago, IL, USA), and horseradish peroxidase (HRP)-conjugated anti-β actin (Cell Signaling Technology). HRP-conjugated anti-rabbit antibody IgG (#1706515; Bio-Rad Laboratories, Hercules, CA, USA) and HRP-conjugated anti-mouse IgG (#1706516; Bio-Rad Laboratories) were used as the secondary antibody. Specific bands were detected using the WEST-ZOL plus Western Blot Detection System (iNtRON Biotechnology, Seongnam, Republic of Korea). CM-H2DCFDA probe and N-acetylcysteine (NAC) were purchased from Sigma-Aldrich (St. Louis, MO, USA).

### 2.2. Plasmid and shRNA

For HBQ1 overexpression, the pLenti6/V5 plasmid was prepared according to the manufacturer’s instructions (V49610; Invitrogen, Carlsbad, CA, USA). Full-length HBQ1 was amplified by polymerase chain reaction (PCR) with an HA tag with a 5′ primer that introduced a BglII site and a 3′ primer that introduced an EcoRI site. HBQ1 coding sequence: 5′-AGA TCT ATG GCG CTG TCC GCG-3′ and 5′-GAA TTC TCA GCG GTA CTC GGA AAC CA-3′. The PCR products were digested with the corresponding enzymes and cloned into the pLenti6/V5 vector. We used pLKO.1-TRC cloning vector (#10878; Addgene, Watertown, MA, USA) to generate shRNA constructs against HBQ1. The individual shHBQ1 RNA targets of human HBQ1 coding sequence: shHBQ1-1:5′-CCG GAA GTT TCC GAG TAC CGC TGA ACT CGA GTT CAG CGG TAC TCG GAA ACT TTT TTT G-3′, shHBQ1-2:5′-CCG GAA GGG TGG GTG GCC GCG GGA TCT CGA GAT CCC GCG GCC ACC CAC CCT TTT TTT G-3′).

### 2.3. Cell Culture and Transfection

Human lung adenocarcinoma cell lines (A549 and A427) were purchased from the American Type Culture Collection (USA). The cells were maintained in RPMI-1640 (Cytiva, Marlborough, MA, USA) supplemented with 10% fetal bovine serum (Cytiva) and 1% penicillin–streptomycin at 37 °C in a humidified 5% CO_2_ incubator. DNA and siRNA were transfected using Lipofectamine 2000 (Invitrogen) according to the manufacturer’s instructions.

### 2.4. Western Blotting

Whole-cell lysates were prepared using radioimmunoprecipitation assay buffer (iNtRON Biotechnology) with a protease inhibitor cocktail (Roche, Basel, Switzerland). Total protein samples were quantified using the Pierce BCA Protein Assay Kit (Thermo Fisher Scientific, Waltham, MA, USA). Equal amounts of cell lysates were resolved via sodium dodecyl-sulfate polyacrylamide gel electrophoresis.

### 2.5. Cell Proliferation Assay

Cells were transfected with HBQ1 plasmid DNA or shHBQ1 for 24 h, trypsinized, and resuspended in a medium. After 48 h, the cells were seeded in 96-well plates at a density of 4 × 10^3^ cells/well. After 72 h of treatment, a mixture of CyQUANT NF Cell proliferation dye reagent and deliverer (Invitrogen) was added to the wells, and the plates were incubated at 37 °C for 30 min. Fluorescence intensity was measured as the ratio of fluorescence at 530 nm to that at 485 nm.

### 2.6. Measurement of ROS Levels

The CM-H2DCFDA probe was employed to assess intracellular ROS production in lung adenocarcinoma cells. These cells were transfected with the specified DNA and HBQ1 shRNA in a 6-well plate. After 48 h, an equal number of cells (1 × 10^5^ cells) were seeded in 96-well plates. Subsequently, lung adenocarcinoma cells were treated with 100 μg/mL of H_2_O_2_ or N-acetyl cysteine (NAC) for 24 h, followed by the addition of 10 μM CM-H2DCFDA for 10 min at 37 °C in the dark. The cells were then washed twice with ice-cold phosphate-buffered saline (PBS). The fluorescence was analyzed using a microplate reader.

### 2.7. In Vivo Tumor Models

Animal experiments were reviewed and approved by the Institutional Animal Care and Use Committee (IACUC) of the National Cancer Center Research Institute. NCCRI is an Association for Assessment and Accreditation of Laboratory Animal Care International (AAALAC International)-accredited facility that abides by the Institute of Laboratory Resources (ILAR) guidelines. Nude mice (BALB/c-nude, five weeks old, females) were purchased from OrientBio (Seongnam, Republic of Korea). The mice were subcutaneously injected with 1.0 × 10^6^ A549 control cells or shHBQ1-transfected cells resuspended in 100 µL of PBS to develop xenograft tumors (*n* = 9 mice per group). Tumor volumes and body weights were measured prior to antibody injection. Tumors were subsequently measured using a caliper, and the volumes were calculated as follows: (W(width)^2^ × L(length)) × 1/2. The mice were sacrificed 35 days after cancer cell injection.

### 2.8. Public Data Analysis

The expression levels of HBQ1 obtained from the Lung Cancer Explorer (https://lce.biohpc.swmed.edu/lungcancer, accessed on 3 July 2023) were used for analysis. Data were obtained from the Beer_2002 and Kabbout_2013 datasets from the Lung Cancer Explorer database. Public datasets for survival analyses of HBQ1 were downloaded from the Kaplan–Meier Plotter (https://kmplot.com/analysis, accessed on 3 July 2023) site. The curve was constructed using Kaplan–Meier-plotter (220807_s_at, Split patients by auto select the best cutoff).

### 2.9. Statistical Analysis

Data are presented as the mean ± standard deviation (SD) from three independent experiments. Statistical analyses were performed with the Student’s *t*-test and the log-rank test. * *p* < 0.05, ** *p* < 0.01, and *** *p* < 0.001 were considered statistically significant.

## 3. Results

### 3.1. Clinical Significance of HBQ1 in Lung Adenocarcinoma

**Figure 1:**
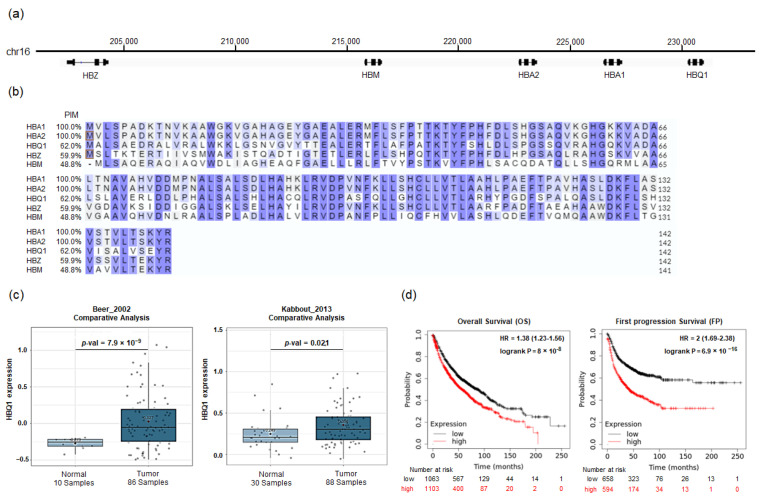
HBQ1 analysis in lung adenocarcinoma. (**a**) Schematic representation of the chromosomal location of the α-globin gene cluster on chromosome 16. (**b**) Multiple sequence alignment of the human α-globin gene cluster, displaying the percent identity matrix (PIM). The PIM shows the percentage of amino acid sequence identities compared to the HBA1 gene (reference sequence). The alignment is colored according to similarity (blue) and initiator methionine (red boxes). (**c**) Box plot comparing specific HBQ1 expression in normal lung tissue (left plot) and lung adenocarcinoma tissue (right plot). (**d**) Survival curve comparing patients with high (red) and low (black) HBQ1 expression in lung adenocarcinoma.

In our investigation of HBQ1 within the human α-globin gene cluster, we compared its chromosome location and amino acid sequence characteristics with the other subunits, namely, HBA1, HBA2, HBZ, and HBM. The analysis revealed that the α-globin genes are located along a 20-kb distal segment of chromosome 16 in the following order from upstream to downstream region: HBZ, HBM, HBA2, HBA1, and HBQ1 (Figure 1a). HBA1, HBA2, HBQ1, and HBZ are each composed of 141 amino acid residues, while HBM is composed of 140 amino acids. Additionally, sequence alignment with the human α-globin protein showed that HBA1 and HBA2 encode the same amino acid sequence despite having different nucleotide sequences. In Figure 1b, HBQ1 exhibited an overall similarity of 62% with HBA1 and HBA2, suggesting that HBQ1 might have a different function compared to the other α-globin gene. We analyzed the association between HBQ1 expression levels and human lung adenocarcinoma and detected a significant upregulation of HBQ1 expression in lung adenocarcinoma tissues compared to normal lung tissues, suggesting its role as an oncogene in lung adenocarcinoma (Figure 1c). Furthermore, we examined the relationship between HBQ1 expression and clinical outcomes in patients with lung cancer. Survival analysis revealed that patients with lung adenocarcinoma with high HBQ1 expression had significant differences in overall survival (OS) and first progression (FP) (Figure 1d).

### 3.2. Role of HBQ1 in Human Lung Adenocarcinoma Cell Proliferation

**Figure 2:**
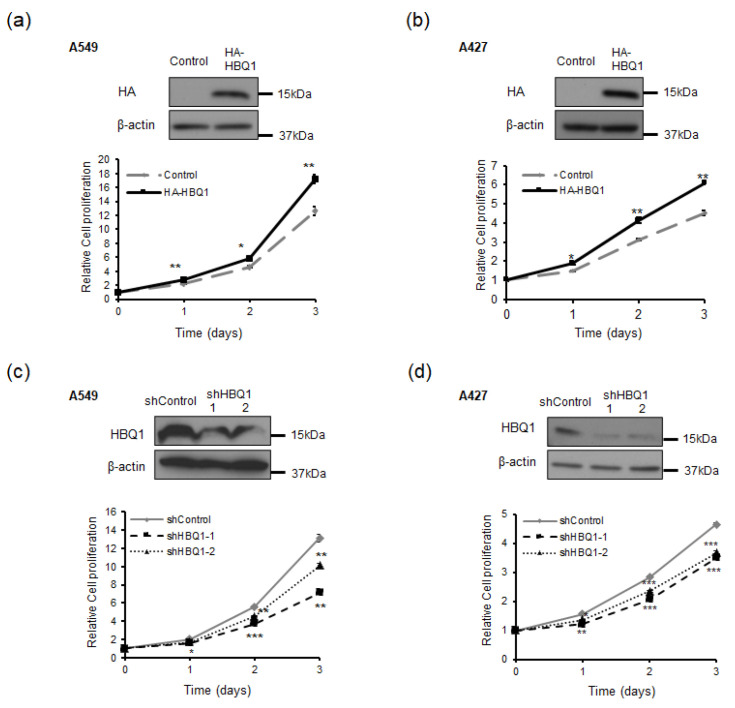
HBQ1 regulates cell proliferation in lung adenocarcinoma cells. (**a**,**b**) A549 and A427 cells were transfected with either the vector control or HA-HBQ1. The overexpression of HBQ1 in A549 and A427 cells was detected through immunoblotting. The effect of HBQ1 overexpression on A549 and A427 cell proliferation was assessed via CyQUANT NF Cell Proliferation Assay. (**c**,**d**) A549 and A427 cells were transfected with either the shControl or shHBQ1. The knockdown of HBQ1 in A549 and A427 cells was detected through immunoblotting. The effect of HBQ1 knockdown on A549 and A427 cell proliferation was assessed via CyQUANT NF Cell Proliferation Assay. Data are expressed as the mean ± SD of different groups of cells from three separate experiments. * *p* < 0.05, ** *p* < 0.01, *** *p* < 0.001.

To investigate the role of HBQ1 in human lung adenocarcinoma cells, we transfected A549 and A427 with either HBQ1 overexpression or HBQ1 knockdown vectors. Changes in HBQ1 expression were confirmed through immunoblotting analysis (Figure 2a–d). Furthermore, we evaluated the effect of HBQ1 expression regulation on cell proliferation. HBQ1 overexpression significantly increased cell proliferation (Figure 2a,b), whereas HBQ1 knockdown resulted in decreased cell proliferation (Figure 2c,d). However, our data do not show any significant impact on the invasion capacity of adenocarcinoma cell lines when HBQ1 expression is regulated.

### 3.3. HBQ1 Promotes Lung Adenocarcinoma Cell Proliferation through Inhibition of ROS Levels

**Figure 3:**
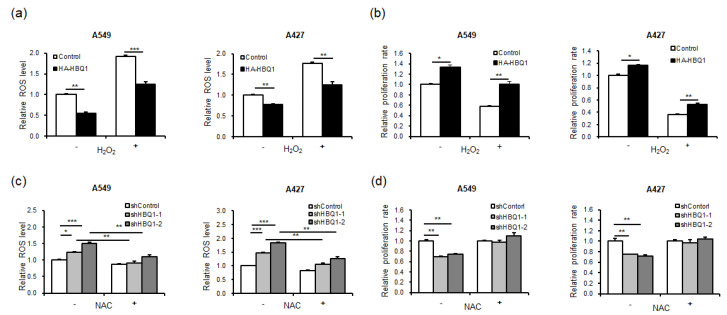
Effect of HBQ1 on intracellular reactive oxygen species (ROS) levels in lung adenocarcinoma cells. (**a**,**b**) The effect of the overexpression or knockdown of HBQ1 on basal cellular ROS levels in lung adenocarcinoma cell lines A549 and A427 cells was assessed using CM-H2DCFDA fluorescent intensity analysis. (**c**,**d**) Intracellular ROS levels and cell proliferation were assessed 24 h after H_2_O_2_ treatment or in the absence of H_2_O_2_ in the Control or HBQ1 overexpression cells. Data are expressed as the mean ± SD of different groups of cells from three separate experiments. * *p* < 0.05, ** *p* < 0.01, *** *p* < 0.001.

We investigated whether HBQ1 expression influences the proliferation of lung adenocarcinoma cells through the regulation of ROS levels. Our results showed that overexpression of HBQ1 led to a decrease in basal ROS levels (Figure 3a), whereas its knockdown led to an increase in basal ROS levels compared to the control group (Figure 3c). This result suggests that HBQ1 has antioxidant properties and can reduce ROS levels under normal conditions. Furthermore, our results demonstrated that HBQ1 overexpression led to a decrease in ROS levels and an increase in cell growth when cells were subjected to H_2_O_2_ treatment, which induces cellular oxidative stress, compared to the control group (Figure 3a,b). Conversely, when HBQ1 was knocked down, higher ROS levels and increased cell growth were observed in cells treated with NAC, an antioxidant, compared to the control group (Figure 3c,d). These findings strongly suggest that HBQ1 functions as an antioxidant, effectively reducing ROS levels and thereby protecting cells from growth inhibition due to oxidative stress.

### 3.4. HBQ1 Knockdown Suppresses Lung Adenocarcinoma Growth In Vivo

**Figure 4:**
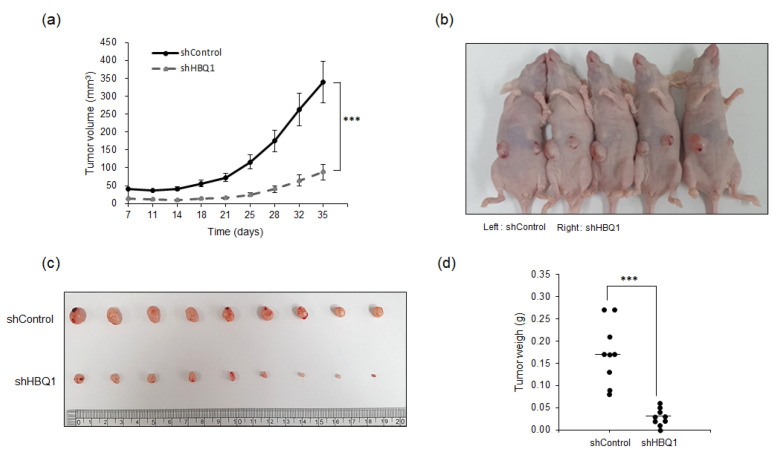
Knockdown of HBQ1 inhibits tumor progression in mouse xenograft models. Stable HBQ1 knockdown A549 cells were subcutaneously injected into nude mice (*n* = 9 mice per group) on 35 days. (**a**) Tumor volumes were calculated at the indicated days after injection. (**b**) Representative image of tumor-bearing mice after injection. (**c**) Representative image of tumors dissected from shControl and shHBQ1 mice. (**d**) Scatter plots represent the weight of independent tumors. The data are presented as the mean ± SD. *** *p* < 0.001.

To further confirm the potential of HBQ1 as a molecular target for lung adenocarcinoma, we established mouse xenograft models by subcutaneously injecting A549 cells with stable HBQ1 knockdown cells. This experiment aimed to verify whether the in vitro findings have relevance in animal models of lung adenocarcinoma in vivo. The results revealed a significant decrease in tumor volume following HBQ1 knockdown using shHBQ1 (Figure 4a). This finding provides strong evidence suggesting that HBQ1 plays a crucial role in promoting the growth of lung cancer, and its inhibition through shHBQ1 effectively suppresses tumor development. Additionally, as shown in Figure 4b–d, a significant reduction in tumor size and weight was observed upon shHBQ1 knockdown, further supporting the involvement of HBQ1 in regulating lung adenocarcinoma growth in vivo. Collectively, our results demonstrate that targeting HBQ1 provides a new therapeutic approach for lung adenocarcinoma treatment. 

## 4. Discussion

In the present study, we demonstrated for the first time that HBQ1 expression is elevated in lung adenocarcinoma tissues compared to normal lung tissues. Furthermore, we examined the relationship between HBQ1 expression and clinical outcomes in patients with lung adenocarcinoma. Kaplan–Meier survival analysis revealed that patients with lung adenocarcinoma with high HBQ1 expression had significant differences in OS and FP. This study provides preliminary evidence that HBQ1, a hemoglobin α subunit, is expressed in lung adenocarcinoma cells.

Recent studies have challenged the long-standing perception that hemoglobin is specifically expressed in erythrocytes [6,7]. These studies have revealed hemoglobin expression in non-erythrocytes, including neurons, retinal cells, alveolar cells, mesangial cells of the kidney, and macrophages [10,11,13,16,26]. In the present study, we performed immunoblotting analysis to investigate the expression of HBQ1 in lung adenocarcinoma cell lines A549 and A427. Notably, our results demonstrated the expression of hemoglobin in these lung adenocarcinoma cell lines. This finding is noteworthy as it demonstrated that HBQ1, which is known to be predominantly expressed in fetal erythroid tissues, is also expressed in lung adenocarcinoma cells.

The main function of hemoglobin in erythrocytes is to transport oxygen from the lungs to the tissues and transport carbon dioxide from the tissues back to the lungs [45,46]. Several studies have been conducted to investigate the functional role of hemoglobin expressed in various cancer types. Notably, the expression of HBA1 and HBB is significantly higher in cervical and bladder cancer tissues than in normal cervix tissues [47,48]. Additionally, the expression of HBB is significantly higher in colorectal cancer [49], and HBB2/HBB is implicated in the regulation of tumorigenesis and metastasis in neuroblastoma [50]. These findings suggest that hemoglobin may be involved in regulating cancer cell progression and development. Based on this evidence, we investigated whether HBQ1 regulates cell proliferation and found that HBQ1 overexpression significantly increased lung adenocarcinoma cell proliferation, whereas HBQ1 knockdown decreased it.

ROS, which are normal byproducts of numerous cellular processes, have been extensively studied in various types of cancers and are considered significant contributors to the development and progression of cancer [34,51,52,53]. Typically, cancer cells exhibit higher basal levels of ROS compared to normal cells due to an imbalance between oxidants and antioxidants [54,55]. ROS plays a dual role in cell metabolism. At low to moderate levels, they act as signal transducers that activate cell proliferation, migration, invasion, and angiogenesis. In contrast, high levels of ROS can cause damage to proteins, nucleic acids, lipids, membranes, and organelles, ultimately leading to cell death [55,56]. Research on the association between hemoglobin and ROS in cancer has been actively pursued and continues to show a growing trend [48,50,57,58]. A previous study has demonstrated that hemoglobin detoxifies highly oxidizing radicals, producing the respective ferric states, which are nontoxic [8,45,46]. Moreover, hemoglobin is more efficient in removing H_2_O_2_ compared to the glutathione peroxidase and glutathione reductase system [59]. β-globin (HBB) suppresses ROS-mediated cytotoxicity, thereby enhancing anchorage-independent cancer cell survival and promoting distant metastasis [48,58,59]. Furthermore, the overexpression of hemoglobin (HBA1 and HBB) in the rat renal mesangial and human hepatocellular cancer cell lines alleviates H_2_O_2_-induced oxidative stress, leading to enhanced cell viability under oxidative stress conditions [22,24]. Although numerous studies have explored ROS-targeted therapy, its application in cancer remains limited. Several factors contribute to this limitation, such as the indiscriminate nature of ROS reactions in all tissues, an abundance of natural antioxidants in the body, potential side effects of certain antioxidant inhibitors, challenges in isolating pure antioxidants from natural sources, and difficulties in accurately assessing the benefits of antioxidant-rich foods. Therefore, novel antioxidants that specifically target cancer cells should be identified. In this regard, HBA1 and HBB present challenging candidates for ROS-targeted cancer therapy due to their known expression in erythrocyte tissues [48,57,58]. In contrast, HBQ1, which has been reported to not be expressed in erythrocyte tissues [31,32], is a promising candidate for ROS-targeted cancer therapy. This specific expression pattern makes HBQ1 an attractive option for reducing ROS levels in lung adenocarcinoma without affecting erythrocyte function. Therefore, targeting non-erythrocyte tissues, where ROS plays a role in cancer, could lead to a more effective approach for addressing oxidative stress-related conditions.

In the present study, HBQ1 acted as an antioxidant, attenuating ROS levels in lung adenocarcinoma cells. These findings suggest that HBQ1 functions as an antioxidant, modulating ROS levels and protecting lung adenocarcinoma cells from proliferation inhibition. In addition, our xenograft results also showed that HBQ1 knockdown inhibited tumor growth in vivo. These results suggest that HBQ1 is pivotal in identifying new antioxidants that can specifically target cancer cells to enhance the effectiveness of ROS-targeted therapy. However, further investigation of the mechanism underlying the role of HBQ1, particularly in its relationship with ROS, is essential to fully explore the therapeutic prospects of HBQ1-based therapies.

## 5. Conclusions

In summary, our study provides evidence of the novel role of HBQ1 in lung adenocarcinoma; HBQ1 is upregulated in lung adenocarcinoma tissues and its high expression is associated with a poor prognosis in patients with this disease. Additionally, our functional experiments revealed that HBQ1 overexpression promoted cell proliferation by reducing basal ROS levels, highlighting its role as a cytoprotective agent against oxidative stress. Conversely, HBQ1 knockdown enhanced ROS levels and inhibited cell proliferation. Furthermore, HBQ1 knockdown effectively suppressed tumor growth in vivo. Taken together, these findings support the crucial involvement of HBQ1 in the development and progression of lung adenocarcinoma and its potential as a therapeutic target.

## Data Availability

Data are contained within the article.
